# Effect of Eu Ions Concentration in Y_2_O_3_-Based Transparent Ceramics on the Electron Irradiation Induced Luminescence and Damage

**DOI:** 10.3390/ma17204954

**Published:** 2024-10-10

**Authors:** Wenhui Lou, Yang Tang, Haohong Chen, Yisong Lei, Hui Lin, Ruijin Hong, Zhaoxia Han, Dawei Zhang

**Affiliations:** 1 Engineering Research Center of Optical Instrument and System, Ministry of Education and Shanghai Key Lab of Modern Optical System, University of Shanghai for Science and Technology, No. 516 Jungong Road, Shanghai 200093, China; 2 Transparent Ceramics Research Center, Shanghai Institute of Ceramics, Chinese Academy of Sciences, Shanghai 201899, China; 3 Institute of Nuclear Physics and Chemistry, China Academy of Engineering Physics, Mianyang 621900, China

**Keywords:** yttrium oxide, transparent ceramics, oxygen vacancy, cathodoluminescence, electron beam irradiation

## Abstract

Eu^3+^-doped Y_2_O_3_-based luminescent materials can be used as a scintillator for electron or high energy β-ray irradiation, which are essential for applications such as electron microscopy and nuclear batteries. Therefore, it is essential to understand their defect mechanisms and to develop materials with excellent properties. In this paper, Y_2_O_3_-based transparent ceramics with different Eu^3+^ doping concentrations were prepared by solid-state reactive vacuum sintering. This series of transparent ceramic samples exhibits strong red emission under electron beam excitation at the keV level. However, color change appears after the high-energy electron irradiation due to the capture of electrons by the traps in the Y_2_O_3_ lattice. Optical transmittance, laser-excited luminescence, X-ray photoelectron spectroscopy (XPS), and other analyses indicated that the traps, or the color change, mainly originate from the residual oxygen vacancies, which can be suppressed by high Eu doping. Seen from the cathodoluminescence (CL) spectra, higher doping concentrations of Eu^3+^ showed stronger resistance to electron irradiation damage, but also resulted in lower emissions due to concentration quenching. Therefore, 10% doping of Eu was selected in this work to keep the high emission intensity and strong radiation resistance both. This work helps to enhance the understanding of defect formation mechanisms in the Y_2_O_3_ matrix and will be of benefit for the modification of scintillation properties for functional materials systems.

## 1. Introduction

Rare earth sesquioxides, such as Y_2_O_3_ and Lu_2_O_3_, have high thermal conductivity and low phonon energy, and are also easily doped with various rare earth ions. Furthermore, their high atomic number and density make them readily absorb ionizing radiation energy and give them strong radiation-blocking ability [1]. These merits make luminescent rare earth ion-activated Y_2_O_3_ and Lu_2_O_3_ sesquioxide materials very promising as phosphors (Eu^3+^:Y_2_O_3_ [2,3,4]), laser gain media (Yb^3+^:Y_2_O_3_ [5,6,7] and Yb^3+^:Lu_2_O_3_ [8,9]), and scintillators (Eu^3+^:Y_2_O_3_ [10]), etc. As a famous red-emitting material, Eu^3+^:Y_2_O_3_ exhibits high brightness, high luminous quantum efficiency, and a long lifetime under ultraviolet or cathode ray excitation [11]. These characteristics make it a promising scintillator for detecting high-energy rays or particles, such as electrons or high-energy β-rays, which have potential applications in electron microscopy, nuclear batteries, industrial non-destructive testing, and other fields [11,12,13]. 

It is known that the growth of a single crystal of Y_2_O_3_ is demanding. Large-sized sesquioxide single crystals are extremely difficult to grow because their melting points are very high (>2400 °C). Furthermore, the phase transformation of Y_2_O_3_ from the monoclinic phase to the hexagonal phase occurs when the temperature is around 2325 °C, which causes changes in the thermal expansion coefficient and density, resulting in lower crystal quality and limiting the growth of large-size crystal. Transparent ceramics exhibit physical and chemical properties, as well as optical characteristics, comparable to those of their single-crystal counterparts. What is more remarkable is that the fabrication of transparent Y_2_O_3_ ceramics can be achieved at a sintering temperature that is lower than the phase transformation temperature. In recent years, to achieve sesquioxide ceramics of high optical quality, numerous sintering techniques have been explored such as vacuum sintering [14,15], hot pressing [16,17], spark plasma sintering [18], and hot isostatic pressing [19,20], among others. All the above methods typically involve the introduction of cationic sintering additives to regulate the grain growth rate [21,22,23,24]. Heterovalent cations, such as Ca^2+^ [25,26], Mg^2+^ [27,28], La^3+^ [29,30], Si^4+^ [26], Zr^4+^ [5,24,31,32], and Hf^4+^ [33], have been extensively tried for the densification of sesquioxide transparent ceramics. However, the incorporation of heterovalent cations typically leads to a charge imbalance within the lattice, thereby generating lattice defects that influence the alteration of material properties. Taking Zr^4+^ as an example, as a heterovalent cation, Zr^4+^ can replace the low-valent cations in the host lattice, resulting in the stimulation the formation of oxygen vacancies in the lattice, which can act as electron-trapping centers [1,34,35]. These oxygen vacancies are capable of capturing electrons, thereby creating deep trap states within the material’s bandgap, which in turn affects the electronic structure and electrical conductivity of the material [5,36]. In scintillation materials, lattice defects may capture electrons or holes, generate color centers, and deteriorate the optical performance of transparent ceramics. They can reduce the radiation resistance of the material. Because radiation resistance is an essential property of scintillators; therefore, both higher radiation resistance and excellent luminescent properties are crucial for scintillation applications. 

Until now, few papers concerning luminescence characteristics and irradiation performance of Eu^3+^:Y_2_O_3_ transparent ceramics under higher-energy electron beams (such as *β*-rays and accelerated electrons) have been published; and the research about the effects of Eu doping concentrations on the defects is also limited. 

In this paper, Y_2_O_3_-based transparent ceramics with different doping concentrations of Eu^3+^ were fabricated by solid-state reactive vacuum sintering. The structural and optical properties of these transparent ceramic samples were investigated. In particular, the impact of Eu^3+^ incorporation into the Y_2_O_3_ lattice on specific defects was examined and discussed in detail using cathodoluminescent spectra, XPS analysis, and other characterizations. 

## 2. Experimental

### 2.1. Materials Preparation

A series of (Eu_x_Y_0.97−x_Zr_0.03_)_2_O_3_ (x = 0.001, 0.01, 0.1, and 0.2) transparent ceramic samples were fabricated by solid-state reactive vacuum sintering. The following oxide powders from Shanghai Aladdin Biochemical Technology Co., Ltd. were used as the raw materials: Y_2_O_3_ (99.99%); Eu_2_O_3_ (99.99%); and ZrO_2_ (99.99%). Polyethylene glycol 400 (PEG-400) was added as a dispersant. The raw powders were mixed and ball-milled in anhydrous ethanol for 16 h. The obtained slurries were dried at 100 °C for 6 h in the oven, screened with a 100-mesh sieve, and calcined at 800 °C for 5 h to remove the organic ingredients. Then, the powders were placed into a steel mold for uniaxial dry-pressing under a pressure of 25 MPa to obtain the tablets. Subsequently, the green bodies were obtained by further pressing the tablets with cold isostatic pressure at 225 MPa for 3 min. Then, the green bodies were sintered in a vacuum furnace at 1850 °C for 5 h, followed by annealing in air at 1350 °C for 7 h. Finally, the (Eu_x_Y_0.97−x_Zr_0.03_)_2_O_3_ (x = 0.001, 0.01, 0.1, and 0.2) transparent ceramic samples were polished on both sides, with a final thickness of 1 mm for each piece.

### 2.2. Characterizations

The crystal structure and phase purity analysis of the samples was performed using an X-ray diffractometer (SmartLab, Rigaku, Tokyo, Japan). X-ray diffraction (XRD) was performed using Cu K_α_ irradiation (λ = 0.15418 nm, step = 0.02°, analysis range of 5–80°), from an X-ray tube operated at 40 kV and 15 mA. The peak indexing for cell parameters and the phase searching were carried out using MDI Jade 6.5 as well as the ICDD PDF2 database attached to the diffractometer. Photoluminescence (PL) spectra were measured by a fluorescence spectrometer (FLS1000, Edinburgh Instruments, Livingston, UK) equipped with a xenon lamp as the excitation light source and a PMT-900 detector (covering a range of 185 nm to 900 nm). The grain size of the sample surface was observed at 10,000× *g* magnification using a scanning electron microscope (SEM, NOVA NANOSEM230, Thermo Fisher Scientific, Waltham, MA, USA). The in-line optical transmittance was measured by an ultraviolet–visible–near-infrared spectrophotometer (SolidSpec-3700i, Shimadzu, Kyoto, Japan) in a wavelength range of 200–800 nm. A cathodoluminescence (CL) spectrometer was established with a cathode ray generator (RELION Industries LLC, RELIOTRON, Bedford, MA, USA) and a spectrometer (QE Pro, Ocean Optics, Dunbury, CT, USA), with the electron beam accelerated at a voltage of 5.0 kV as the excitation source. The thermoluminescence (TL) curve was measured by a TL spectrometer (TOSL-3DS, Rongfan, Guangzhou, China) in a temperature range of 300–575 K. Before the TL tests, the samples were irradiated by X-ray from a tungsten tube with 50 kV and 80 μA. The irradiation dose rate was 0.5 Gy/min and the total irradiation time was 5 min. The heating rate for the TL spectra was 1 K/s, and the spectral measurement utilized a high-sensitivity CCD spectral measurement system. The CCD’s wavelength measurement range was 300–850 nm. XPS analysis was performed on an XPS spectrometer (Thermo Scientific K-Alpha, Thermo Fisher Scientific, Waltham, MA, USA) using Al K_α_ (hυ = 1486.6 eV) as the X-ray excitation source. Additionally, Avantage™ v5.984 software was used to fit and analyze the XPS data.

## 3. Results and Discussion

**Figure 1:**
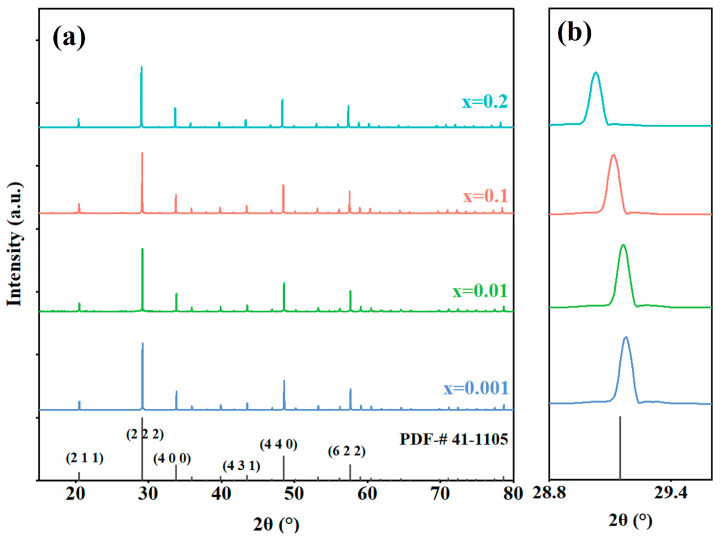
XRD *θ*-2*θ* scanning patterns (**a**); and enlarged view in the range of 28–30° (**b**) of the (Eu_x_Y_0.97−x_Zr_0.03_)_2_O_3_ (x = 0.001, 0.01, 0.1, and 0.2) ceramic samples.

**Figure 2:**
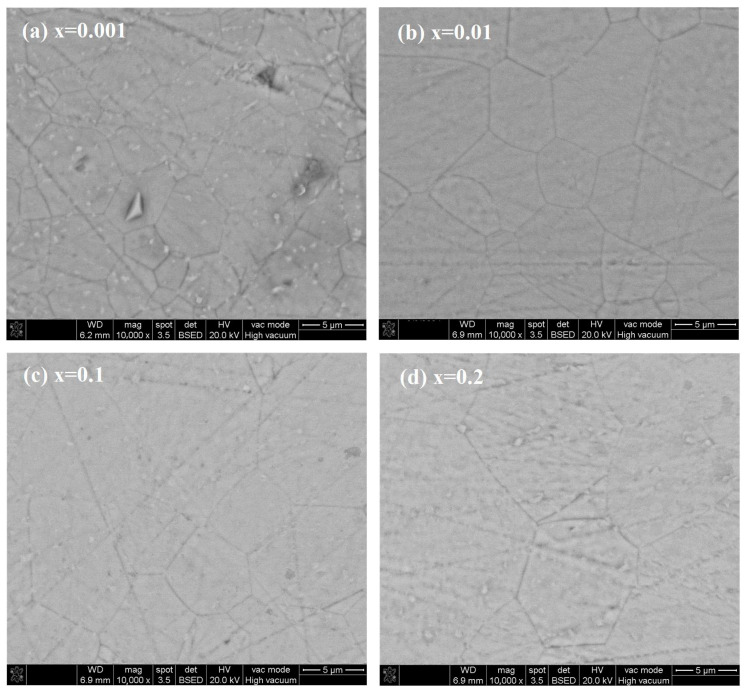
SEM surface morphology of the (Eu_x_Y_0.97−x_Zr_0.03_)_2_O_3_ (x = 0.001, 0.01, 0.1, and 0.2) ceramic samples: (**a**) x = 0.001; (**b**) x = 0.01; (**c**) x = 0.1; and (**d**) x = 0.2.

X-ray diffraction patterns of (Eu_x_Y_0.97−x_Zr_0.03_)_2_O_3_ (x = 0.001, 0.01, 0.1, and 0.2) transparent ceramic samples are presented in Figure 1. It can be observed that all the diffraction peaks of each sample correspond to those of the Y_2_O_3_ standard card (PDF#41-1105); no extra peaks are observed. In Figure 1b, the main XRD peaks have been amplified for detailed observation and are set to a reference peak with a relative intensity of 100%. It can be clearly seen in the figure that with the increase in Eu^3+^ concentration, the position of the diffraction peak gradually moves to a lower angle. With the increase of Eu^3+^ concentration from 0.1% to 20%, the grain size gradually increased from 10.59 Å to 10.64 Å, according to the analysis of XRD data with MDI Jade 6.5. All the above results prove that the Eu^3+^ was doped into the Y_2_O_3_ lattice, forming a solid solution [2,37,38,39]. As shown in Figure 2, all samples exhibit an almost pore-free dense microstructure. With the increase in Eu^3+^ concentration, the grain size of the Y_2_O_3_-based transparent ceramic samples also increased gradually.

**Figure 3:**
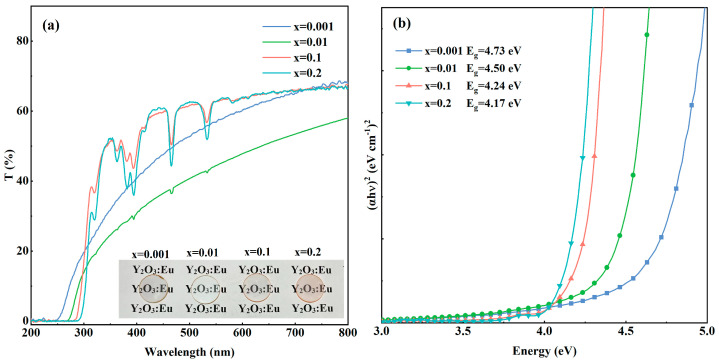
Optical transmission spectra (**a**); and the calculated optical bandgap energy (**b**) of the (Eu_x_Y_0.97−x_Zr_0.03_)_2_O_3_ (x = 0.001, 0.01, 0.1, and 0.2) ceramic samples.

The in-line optical transmittance curves of the ceramic samples are shown in Figure 3a. When the doping concentration of Eu^3+^ is high (10% and 20%), the optical transmittance of the samples at 612 nm is about 64% and the distinct absorption bands peak are at 394 nm (^7^F_0_→^5^L_6_), 467 nm (^7^F_0_→^5^D_2_), and 533 nm (^7^F_1_→^5^D_1_), respectively [37,38,39]. In the range of 250–300 nm, the absorption edges of (Eu_x_Y_0.97−x_Zr_0.03_)_2_O_3_ (x = 0.001, 0.01, 0.1, and 0.2) transparent ceramic samples change with the variation in Eu^3+^ concentrations. The bandgap energy of the samples can be calculated using the Tauc plot method [40]. The equation is as follows:

(1) $${\left(\alpha h\upsilon \right)}^{\frac{1}{n}}=B(h\upsilon -E_{g})$$

where *α* is the absorption coefficient, *h* is Planck’s constant, *υ* is incident photon frequency, *E_g_* is the bandgap energy, and n is the index that determines the type of transition. Y_2_O_3_ is an indirect bandgap material (*n* = 2). Figure 3b shows the bandgap energy plots of the samples. The doping can introduce additional energy bands into the original forbidden band of the matrix (Y_2_O_3_ in this case), resulting in a lower bandgap energy. Thus, the lower bandgap energy can be attributed to the increased Eu concentration.

**Figure 4:**
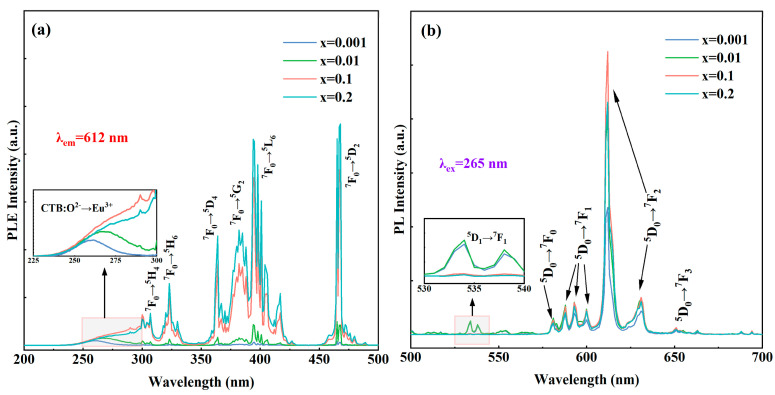
PLE (**a**); and PL (**b**) spectra of the (Eu_x_Y_0.97−x_Zr_0.03_)_2_O_3_ (x = 0.001, 0.01, 0.1, and 0.2) ceramic samples.

Figure 4a shows the PLE spectra of (Eu_x_Y_0.97−x_Zr_0.03_)_2_O_3_ (x = 0.001, 0.01, 0.1, and 0.2) ceramics with the 612 nm red emission monitored, where each spectrum shows a broad band at 265 nm and narrow bands at 395 nm and 466 nm. The excitation peak at 265 nm is caused by the charge transfer band (CTB) between O^2−^ and Eu^3+^. And the narrow band excitations are attributed to the Eu^3+^ transitions, as follows: ^7^F_0_→^5^D_4_ (364 nm); ^7^F_0_→^5^D_3_ (417 nm); ^7^F_0_→^5^L_6_ (395 nm); and ^7^F_0_→^5^D_2_ (466 nm) transitions [29,31,32]. In Y_2_O_3_, Eu^3+^ replaces Y^3+^ to form complex ions with O^2−^ in the lattice. When the Eu^3+^ doping concentration exceeds 1%, the CTB peak gradually broadens and intensifies, which may be due to the decrease in the bandgap with heavily doping of the Eu^3+^ ions to increase the possibility of charge transfer. Figure 4b shows the PL spectra of (Eu_x_Y_0.97−x_Zr_0.03_)_2_O_3_ (x = 0.001, 0.01, 0.1, and 0.2) ceramics under the 265 nm excitation. The most intense emission peaks are located near 612 nm. The emission peak at 612 nm corresponds to the Eu^3+^: ^5^D_0_→^7^F_2_ electric dipole transition [21,22,23,24]. In the Y_2_O_3_ lattice, Eu^3+^ ions occupy the C_2_ and C_3i_ (S_6_). Eu^3+^ at the C_2_ position is more likely to promote the Eu^3+^: ^5^D_0_→^7^F_2_ transition, corresponding to the position of the strongest emission peak of Eu^3+^:Y_2_O_3_ [21,41,42,43,44,45,46]. Mayrinck et al. proposed that the Eu^3+^: ^5^D_0_→^7^F_2_ transition in Y_2_O_3_ can be referred to as a hypersensitive transition [2]. As seen in Figure 4b, with the increase in the Eu^3+^-doping concentration, the emission peak intensity at 612 nm under the 265 nm excitation gradually increases. This is attributed to most of the Eu^3+^ ions occupying the C_2_ site of the Y_2_O_3_ lattice, causing the ^5^D_0_→^7^F_2_ transition to be dominant [3,21,42]. 

**Figure 5:**
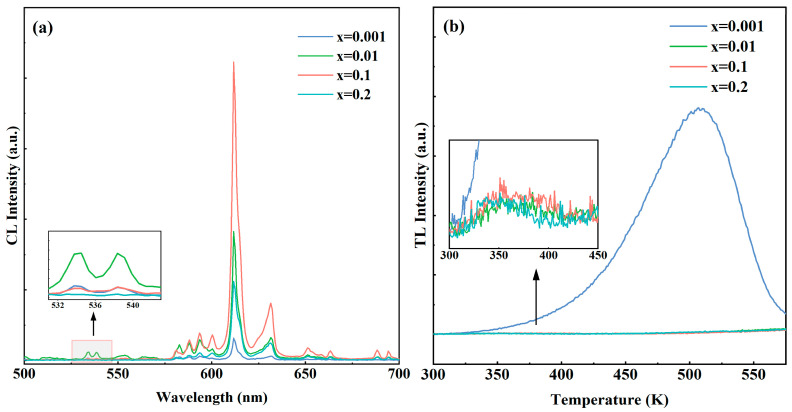
CL (**a**); and TL (**b**) spectra of (Eu_x_Y_0.97−x_Zr_0.03_)_2_O_3_ (x = 0.001, 0.01, 0.1, and 0.2) ceramic samples.

Figure 5a shows the CL spectra of (Eu_x_Y_0.97−x_Zr_0.03_)_2_O_3_ (x = 0.001, 0.01, 0.1, and 0.2) ceramic samples with Eu^3+^ ion-doping concentrations of 0.1%, 1%, 10%, and 20% under low voltage (5.0 kV) excitation; the characteristic emission peaks of Eu^3+^ can be seen in the figures. The increase in Eu content significantly enhances the characteristic transition intensity of ^5^D_0_→^7^F_2_ [24,42]. When the Eu^3+^doping concentration is 10%, the luminescence intensity at 612 nm is the highest. With the increase in Eu^3+^ doping concentration, the average distance between adjacent Eu^3+^ ions decreases; thus, non-radiative decay can be made easier since the enhanced energy transfer between the Eu^3+^ cations may also cause energy loss to luminescence quenching centers such as defects. Then, the typical concentration quenching occurs at higher concentrations [24,37,42,46]. At the same time, it can be seen that there is an emission peak in the range of 530–540 nm in Figure 4b and Figure 5a, which corresponds to the Eu^3+^: ^5^D_1_→^7^F_1_ transition. When the Eu^3+^ doping concentration is 1%, the emission peak intensity here reaches a maximum. When the doping concentration exceeds 10%, concentration quenching occurs and the emission intensity decreases significantly. The decrease in the ^5^D_1_→^7^F_1_ transition intensity occurs because the ^5^D_1_ level is a higher energy level [46,47]. When the Eu content increases, the rate of non-radiative decay from ^5^D_1_→^5^D_0_ is greater than the rate of ^5^D_1_→^7^F_1_ transition, and non-radiative transition dominates. Figure 5b shows the TL curves of the (Eu_x_Y_0.97−x_Zr_0.03_)_2_O_3_ (x = 0.001, 0.01, 0.1, and 0.2) ceramic samples. With the temperature increased, the peak position and intensity (or maximum) indicate the depth and the amount of the defects in the material, respectively. It can be observed that the sample with a doping concentration of 0.1% has a pronounced TL peak at 508 K. The samples with higher doping concentrations (1 and 10%) only have a very small TL peak in the range of 300–450 K. Therefore, there are fewer and shallower defects in the ceramics with higher Eu^3+^ concentrations, which also agrees with the following conclusion on the inhibitory effect of heavily Eu^3+^ doping on traps.

**Figure 6:**
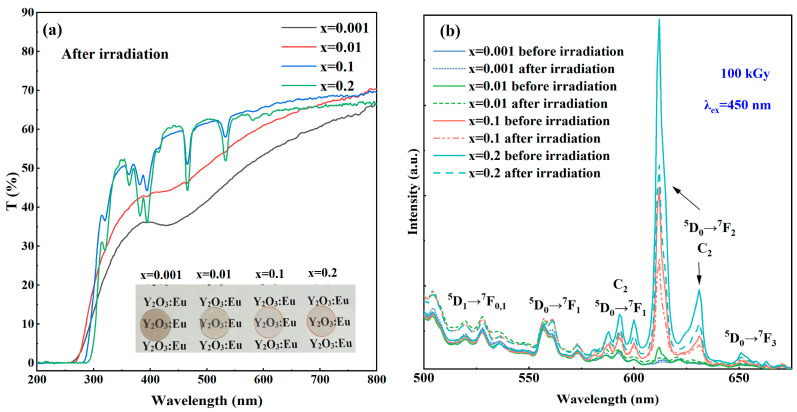
Transmittance (**a**); and PL (**b**) spectra of Eu^3+^:Y_2_O_3_ ceramic samples under the 450 nm excitation after accelerated electron irradiation.

In order to study the defects and their effects on optical properties, the irradiation damage of this series of samples was tested with high-energy electrons. Figure 6a illustrates a photograph of the sample after accelerated electron irradiation at 100 kGy. The color of all samples changed under the irradiation of the electron beam. It originated from the defects in the ceramic samples, which captured electrons or holes to form color centers. The color of the Y_2_O_3_ ceramic with the lowest Eu concentration changed significantly after the irradiation. 

There are oxygen vacancies inside Y_2_O_3_; the Schottky defect reaction equation in Y_2_O_3_ is as follows [48]:

(2) $$Y_{2}O_{3}\to 2V_{Y}^{\prime \prime \prime }+3V_{O}^{\cdot \cdot }$$

In the electron-irradiated (Y_0.999_Eu_0.001_)_2_O_3_ ceramic sample, the brown color indicates that there are a large number of oxygen vacancies in the sample with an Eu^3+^ content of only 0.1%. When the concentration of Eu^3+^ is deficient, the electrons generated by the conduction band are captured mainly by oxygen vacancies during irradiation, resulting in severe discoloration. When the doping concentration of Eu^3+^ is higher, due to the higher electronegativity of the Eu–O bond than that of Y–O, it is reasonable to expect that oxygen ions can be bonded more tightly in the lattice to suppress the production of oxygen vacancies. Furthermore, a part of Eu^3+^ will also capture electrons and compete with oxygen vacancies. Both can thus inhibit color generation in the samples. Therefore, the doping of Eu^3+^ has a competitive or inhibitory effect on the capture of electrons by oxygen vacancies. In order to further explore the reasons for the color change in ceramic samples after irradiation and the inhibition mechanism of Eu^3+^ doping concentrations on the internal defects of ceramic samples, the optical transmittance spectra of the samples and the emission spectra at 450 nm were studied. Figure 6a shows the transmission curves of the irradiated samples. It was found that when the Eu^3+^ doping concentration was less than 1%, a prominent absorption peak [1,5] appeared in the range of 380–480 nm, along with a lower base line in comparison with that seen in Figure 3a, which can be attributed to the valence band electrons being excited and trapped into the defect level under high-energy electron irradiation. As the electrons in the defect state are excited to a higher energy level, they will absorb light of a specific wavelength and form an absorption peak, which tends to disappear with the increase in the Eu^3+^ concentration. The PL spectra of the irradiated samples excited by 450 nm blue light are shown in Figure 6b. The luminescence intensity of the samples decreased after irradiation, which agrees with the color change and lower optical transmittance in the emission wavelength range.

**Figure 7:**
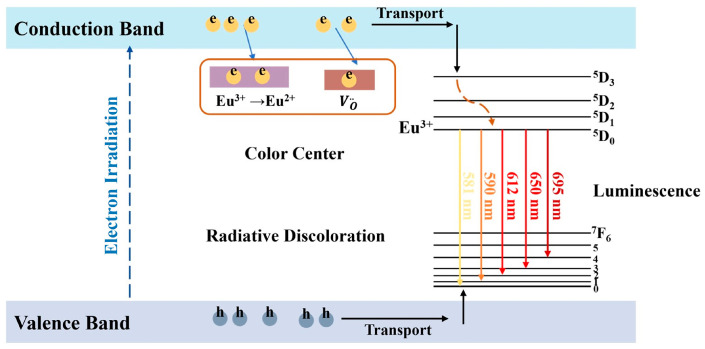
Schematic diagram to illustrate the mechanism of the electron beam irradiation induced color center as well as the red emission.

In all, as shown in Figure 7, the radiochromic process of Eu^3+^:Y_2_O_3_ ceramics can be explained as follows: When exposed to radiation, the residual oxygen vacancies in the Y_2_O_3_ lattice capture electrons from the conduction band to produce color centers. When the Eu^3+^ concentration is very high, not only the residual oxygen vacancies decrease, but also some of the electrons in the conduction band are captured by Eu^3+^, causing the Eu^3+^ to convert to Eu^2+^. Consequently, the number of electrons captured by the oxygen vacancies is reduced. Therefore, the color of the sample with a high Eu^3+^ concentration after the irradiation is lighter than that of the samples with a low Eu^3+^ concentration. This indicates that Eu^3+^:Y_2_O_3_ ceramics with a high Eu^3+^ concentration exhibit stronger irradiation damage resistance. 

**Figure 8:**
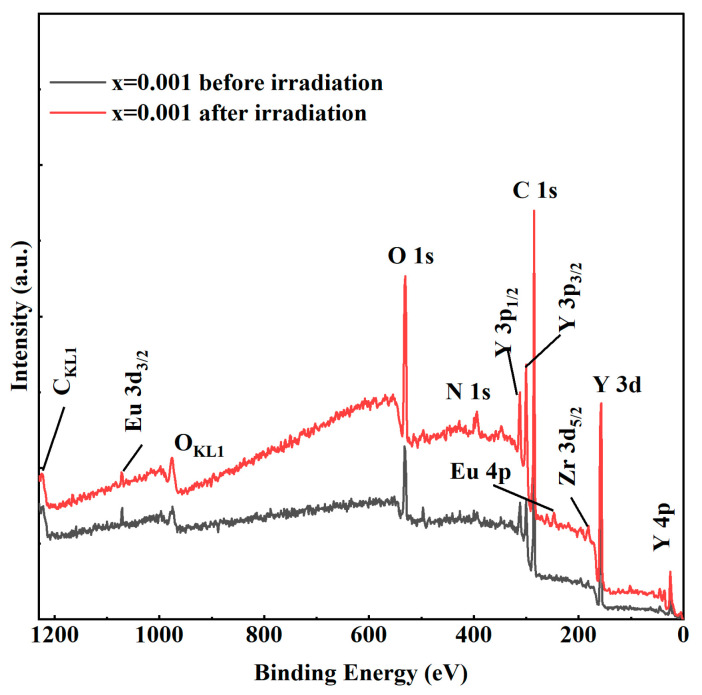
XPS comparison for 0.1% Eu^3+^:Y_2_O_3_ ceramic before and after irradiation.

**Figure 9:**
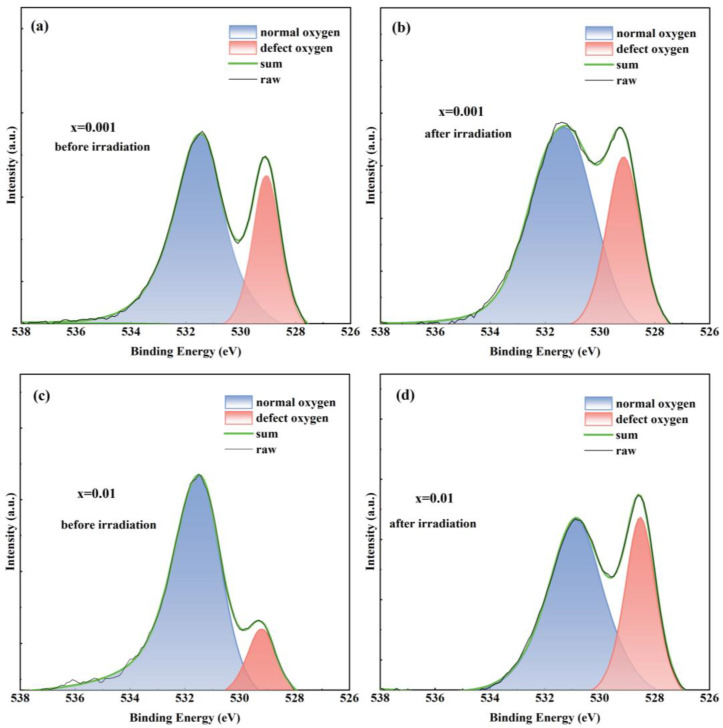

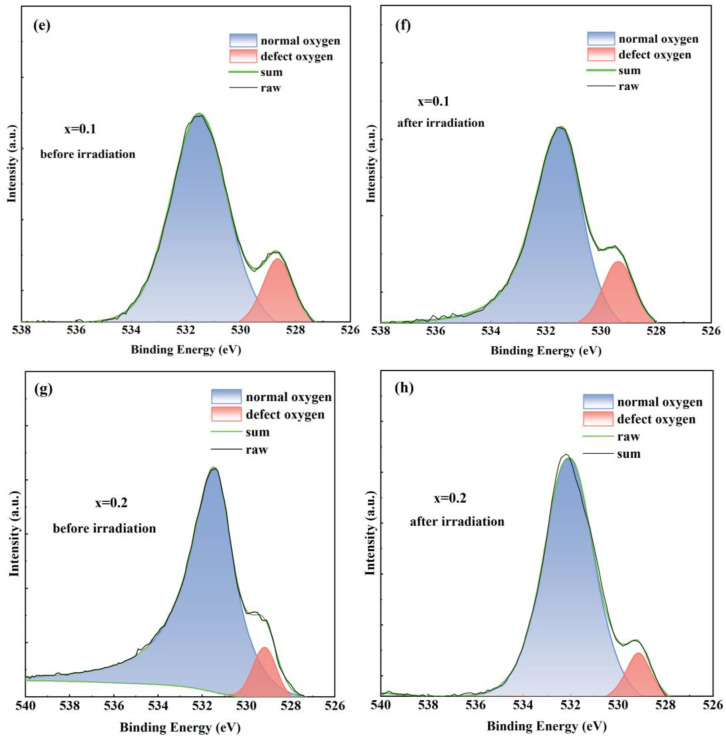
O 1s XPS signals fitting of (Eu_x_Y_0.97−x_Zr_0.03_)_2_O_3_ (x = 0.001, 0.01, 0.1, and 0.2) ceramic samples before and after irradiation: (**a**,**b**) x = 0.001; (**c**,**d**) x = 0.01; (**e**,**f**) x = 0.1; and (**g**,**h**) x = 0.2.

Figure 8 shows the XPS of Eu^3+^:Y_2_O_3_ ceramic samples with a Eu^3+^ doping concentration of 0.1% before and after irradiation. The XPS signal intensity of the irradiated sample is enhanced, which is attributed to the differences in the surface before and after the irradiation. After irradiation with high-energy charged particles (electrons, in this case), the surface structure can vary or be destroyed due to the breaking of chemical bonds by the absorption of higher energy. A more stable surface indicates higher radiation resistance. To study the effect of Eu concentrations on irradiation damage, the high-resolution O1s spectra were recorded and decomposed, as shown in Figure 9.

The XPS spectra of O1s before irradiation are shown in Figure 9a, Figure 9c, Figure 9e and Figure 9g, while Figure 9b, Figure 9d, Figure 9f and Figure 9h display the O1s XPS signals of samples after irradiation. All the O1s spectral signals of the samples can be well decomposed into two peaks. According to the block state in the centimeter-sized examples, the peak with the higher binding energy at 531.40 eV corresponds to normal oxygen ions similar to those found in the bulk of the sample, and the peak at 529.05 eV corresponds to the oxygen ions with a coordinated surrounding that is farther away from the normal oxygen ions at the surface [21,48,49], here noted as “defect oxygen ions”. 

After the destruction of the surface using high-energy charged particles, it can be seen that the normal oxygen content after irradiation is significantly reduced compared with that before irradiation, which indicates that destruction occurred on the surface during the irradiation. This observation is also consistent with the color change discussed earlier. By comparing the reduction in normal oxygen before and after irradiation, the increase in Eu^3+^ concentration inhibited the reduction in normal oxygen, as shown in Table 1. Therefore the samples with higher Eu^3+^ doping concentrations have more normal oxygen ions, indicating an enhanced ability to resist irradiation damage. This further confirms the inhibitory effect of Eu^3+^ on electron irradiation damage, implying greater surface stability.

## 4. Conclusions

In this paper, (Eu_x_Y_0.97−x_Zr_0.03_)_2_O_3_ (x = 0.001, 0.01, 0.1, and 0.2) transparent ceramics were prepared by solid-state reactive vacuum sintering, with ZrO_2_ as the sintering aid. The coloration mechanism during high-energy electron irradiation was studied. The residual oxygen vacancies in Y_2_O_3_ captured electrons to produce color centers, resulting in a reddish-brown color. With the increase in Eu^3+^ concentration, the color generated by irradiation gradually became lighter, and the electron-beam irradiation resistance was improved. Eu doping at 10% resulted in both a high irradiation hardness and a strong red emission. This work improves the understanding of defect formation and suppression mechanisms, which could be beneficial for Y_2_O_3_-based scintillator applications such as high energy *β*-ray-based nuclear batteries and electron imaging.

## Figures and Tables

**Table 1:** The proportion of normal oxygen content before and after irradiation.

	x = 0.001	x = 0.01	x = 0.1	x = 0.2
Before irradiation	71.48%	84.12%	84.11%	91.04%
After irradiation	54.38%	62.27%	80.64%	87.72%

## Data Availability

The raw data supporting the conclusions of this article will be made available by the authors on request.

## References

[1] Liu X., Zheng J., Xu Z., Du W., Zuo Y., Guo H., Jing W., Feng H. (2022). X-ray induced coloration behavior of Lu_2_O_3_:Eu transparent ceramics and the impact of ZrO_2_ and HfO_2_ sintering additives. Opt. Mater..

[2] Mayrinck C.D., Santos D.P., Ribeiro S.J.L., Schiavon M.A., Ferrari J.L. (2014). Reassessment of the potential applications of Eu^3+^-doped Y_2_O_3_ photoluminescent material in ceramic powder form. Ceram. Int..

[3] Xu H., Yu W., Pan K., Wang G., Zhu P. (2021). Confinement and antenna effect for ultrasmall Y_2_O_3_:Eu^3+^ nanocrystals supported by MOF with enhanced near-UV light absorption thereby enhanced luminescence and excellently multifunctional applications. Nano Res..

[4] Prasanna Kumar J.B., Ramgopal G., Vidya Y.S., Anantharaju K.S., Daruka Prasad B., Sharma S.C., Prashantha S.C., Premkumar H.B., Nagabhushana H. (2015). Bio-inspired synthesis of Y_2_O_3_:Eu^3+^ red nanophosphor for eco-friendly photocatalysis. Spectrochim. Acta Part A Mol. Biomol. Spectrosc..

[5] Wang J., Yin D., Ma J., Liu P., Wang Y., Dong Z., Kong L.B., Tang D. (2019). Pump laser induced photodarkening in ZrO_2_-doped Yb:Y_2_O_3_ laser ceramics. J. Eur. Ceram. Soc..

[6] Zhidkov I., Kukharenko A., Maksimov R., Finkelstein L., Cholakh S., Osipov V., Kurmaev E. (2019). Optical Transparency and Local Electronic Structure of Yb-Doped Y_2_O_3_ Ceramics with Tetravalent Additives. Symmetry.

[7] Ning J., Wang D., Luo Z.L., Dong L.B., Kong D. (2017). Low-level sintering aids for highly transparent Yb:Y_2_O_3_ ceramics. J. Alloys Compd..

[8] Basyrova L., Loiko P., Maksimov R., Shitov Serres V., Griebner U., Petrov V., Aguiló M., Díaz F., Mateos X. (2021). Comparative study of Yb:Lu_3_Al_5_O_12_ and Yb:Lu_2_O_3_ laser ceramics produced from laser-ablated nanopowders. Ceram. Int..

[9] Dong L., Ma M., Jing W., Xu T., Bin K., Guo R. (2019). Synthesis of highly sinterable Yb:Lu_2_O_3_ nanopowders via spray co-precipitation for transparent ceramics. Ceram. Int..

[10] García-Muñoz M., Jimenez-Rey D., García-Lopez J., Zurro B., Baciero A., Fahrbach H., McCarthy K.J. (2011). Characterization of scintillator screens for suprathermal ion detection in fusion devices. J. Instrum..

[11] Weber M.J. (2002). Inorganic scintillators: Today and tomorrow. J. Lumin..

[12] Nikl M., Yoshikawa A. (2015). Recent R&D Trends in Inorganic Single-Crystal Scintillator Materials for Radiation Detection. Adv. Opt. Mater..

[13] Touš J., Blažek K., Pína L., Sopko B. (2010). High-resolution imaging of biological and other objects with an X-ray digital camera. Appl. Radiat. Isot..

[14] Liu H., Zhu L., Tan X., Xu X., Zhang G., Jian Y., Dai M., Chen J., Lin H. (2023). Fabrication and properties of highly transparent Er_2_O_3_ ceramics with ZrO_2_ additive by vacuum sintering. Ceram. Int..

[15] Li X., Mao X., Feng M., Xie J., Jiang B., Zhang L. (2016). Optical absorption and mechanism of vacuum-sintered ZrO_2_-doped Y_2_O_3_ ceramics. J. Eur. Ceram. Soc..

[16] Huang S., Li Q., Wang Z., Cheng X., Wen H. (2017). Effect of sintering aids on the microstructure and oxidation behavior of hot-pressed zirconium silicate ceramic. Ceram. Int..

[17] Balabanov S., Permin D., Evstropov T., Andreev P., Basyrova L., Camy P., Baranov M., Mateos X., Loiko P. (2021). Hot pressing of Yb:Y_2_O_3_ laser ceramics with LiF sintering aid. Opt. Mater..

[18] Lee J., Kim B., Jang B. (2020). Non-uniform sintering behavior during spark plasma sintering of Y_2_O_3_. Ceram. Int..

[19] Fan L., Jiang M., Lin D., Zhou D., Shi Y., Wu Y., Yao H., Xu F., Xie J., Lei F. (2017). Densification of cerium-doped lutetium oxyorthosilicate scintillation ceramics by hot isostatic pressing. J. Alloys Compd..

[20] Wang J., Ma J., Zhang J., Liu P., Luo D., Yin D., Tang D., Kong L. (2017). Yb:Y_2_O_3_ transparent ceramics processed with hot isostatic pressing. Opt. Mater..

[21] Kumar Y., Pal M., Herrera M., Mathew X. (2016). Effect of Eu ion incorporation on the emission behavior of Y_2_O_3_ nanophosphors: A detailed study of structural and optical properties. Opt. Mater..

[22] Yavetskiy R., Dobrotvorskaya M., Doroshenko A., Tolmachev A., Petrusha I., Turkevich V., Tomala R., Hreniak D., Strek W., Baumer V. (2018). Fabrication and luminescent properties of (Y_0.99_Eu_0.01_)_2_O_3_ transparent nanostructured ceramics. Opt. Mater..

[23] Wang Z., Zhou G., Zhang J., Qin X., Wang S. (2017). Luminescence properties of Eu^3+^-doped Lanthanum gadolinium hafnates transparent ceramics. Opt. Mater..

[24] Orekhova K., Tomala R., Zamoryanskaya M. (2021). The study of composition, structure and cathodoluminescent features of YAG:Eu^3+^ nanoceramics. Excitation capture efficiency of Eu^3+^ energy levels. J. Alloys Compd..

[25] Rétot H., Blahuta S., Bessière A., Viana B., LaCourse B. (2011). Mattmann, Improved scintillation time response in (Lu_0.5_Gd_0.5_)_2_O_3_:Eu^3+^ compared with Lu_2_O_3_:Eu^3+^ transparent ceramics. J. Phys. D Appl. Phys..

[26] Chaika M.A., Mancardi G., Vovk O.M. (2020). Influence of CaO and SiO_2_ additives on the sintering behavior of Cr,Ca:YAG ceramics prepared by solid-state reaction sintering. Ceram. Int..

[27] Chen S., Yang S., Chen L., Ma Z., Chen J., Guo W. (2023). MgO-Y_2_O_3_:Eu composite ceramics with high quantum yield and excellent thermal performance. J. Eur. Ceram. Soc..

[28] Itatani K., Tsujimoto T., Kishimoto A. (2006). Thermal and optical properties of transparent magnesium oxide ceramics fabricated by post hot-isostatic pressing. J. Eur. Ceram. Soc..

[29] Yi Q., Zhou S., Teng H., Lin H., Hou X., Jia T. (2012). Structural and optical properties of Tm:Y_2_O_3_ transparent ceramic with La_2_O_3_, ZrO_2_ as composite sintering aid. J. Eur. Ceram. Soc..

[30] Zhu L.-L., Park Y.-J., Gan L., Go S.-I., Kim H.-N., Kim J.-M., Ko J.-W. (2017). Effects of ZrO_2_-La_2_O_3_ co-addition on the microstructural and optical properties of transparent Y_2_O_3_ ceramics. Ceram. Int..

[31] Hou X., Zhou S., Li W., Li Y. (2010). Study on the effect and mechanism of zirconia on the sinterability of yttria transparent ceramic. J. Eur. Ceram. Soc..

[32] Sójka M., Kulesza D., Bolek P., Trojan-Piegza J., Zych E. (2019). Tracing mechanism of optically and thermally stimulated luminescence in Lu_2_O_3_:Tb,M (M = Hf, Zr, Ti) ceramic storage phosphors. J. Rare Earths.

[33] Subbarao E.C., Sutter P.H., Hrizo J. (1965). Defect Structure and Electrical Conductivity of ThO_2_-Y_2_O_3_ Solid Solutions. J. Am. Ceram. Soc..

[34] Jolley A., Cohn G., Hitz G., Wachsman E. (2015). Improving the ionic conductivity of NASICON through aliovalent cation substitution of Na_3_Zr_2_Si_2_PO_12_. Ionics.

[35] Shukla J., Saxena P., Joshi P., Joshi P., Mishra A. (2023). Impact of aliovalent ions doping on structural and electrical characteristics of YMnO_3_ ceramic. Appl. Phys. A.

[36] Izmailov R., Strand J., Larcher L., O’Sullivan B., Shluger A., Afanas’ev V. (2021). Electron trapping in ferroelectric HfO_2_. Phys. Rev. Mater..

[37] Nakauchi D., Okada G., Koshimizu M., Yanagida T. (2016). Storage luminescence and scintillation properties of Eu-doped SrAl_2_O_4_ crystals. J. Lumin..

[38] Lu B., Li J., Suzuki T.S., Estili M., Liu W., Sun X., Sakka Y. (2015). Controlled Synthesis of Layered Rare-Earth Hydroxide Nanosheets Leading to Highly Transparent (Y_0.95_Eu_0.05_)_2_O_3_ Ceramics. J. Am. Ceram. Soc..

[39] Podowitz S.R., Gaumé R., Feigelson R.S. (2010). Effect of Europium Concentration on Densification of Transparent Eu:Y_2_O_3_ Scintillator Ceramics Using Hot Pressing. J. Am. Ceram. Soc..

[40] Zhong H., Pan F., Yue S., Qin C., Hadjiev V., Tian F., Liu X., Lin F., Wang Z., Bao J. (2023). Idealizing Tauc Plot for Accurate Bandgap Determination of Semiconductor with Ultraviolet–Visible Spectroscopy: A Case Study for Cubic Boron Arsenide. J. Phys. Chem. Lett..

[41] Chaonan W., Weiping Z., Min Y. (2009). Preparation and spectroscopic properties of Y_2_O_3_:Eu^3+^ nanopowders and ceramics. J. Alloys Compd..

[42] Kowalczyk M., Kaczkan M., Majchrowski A., Malinowski M. (2019). Short-wavelength luminescence of Eu^3+^-doped KGd(WO_4_)_2_ crystals. Opt. Mater..

[43] Fu Z., Zhou S., Pan T., Zhang S. (2007). Preparation and luminescent properties of cubic Eu^3+^:Y_2_O_3_ nanocrystals and comparison to bulk Eu^3+^:Y_2_O_3_. J. Lumin..

[44] Shivaramu N.J., Lakshminarasappa B.N., Nagabhushana K.R., Singh F., Swart H.C. (2017). Synthesis, thermoluminescence and defect centres in Eu^3+^ doped Y_2_O_3_ nanophosphor for gamma dosimetry applications. Mater. Res. Express.

[45] Den Engelsen D., Fern G.R., Ireland T.G., Harris P.G., Hobson P.R., Lipman A., Dhillon R., Marsh P.J., Silver J. (2016). Ultraviolet and blue cathodoluminescence from cubic Y_2_O_3_ and Y_2_O_3_: Eu^3+^ generated in a transmission electron microscope. J. Mater. Chem. C.

[46] Zhou S., Duan C., Yin M., Zhang S., Wang C. (2019). High-sensitive optical temperature sensing based on ^5^D_1_ emission of Eu^3+^ in YVO_4_. J. Alloys Compd..

[47] Singh A.K., O’Donnell K., Edwards P.R., Cameron D., Lorenz K., Kappers M.J., Boćkowski M., Yamaga M., Prakash R. (2017). Luminescence of Eu^3+^ in GaN(Mg, Eu): Transitions from the ^5^D_1_ level. Appl. Phys. Lett..

[48] Chen P., Chen I. (1996). Grain Boundary Mobility in Y_2_O_3_: Defect Mechanism and Dopant Effects. J. Am. Ceram. Soc..

[49] Ren Y., Li X., Zhang Z., Mu H., Zhu Q., Fu Z., Sun X. (2023). Effects of Zr^4+^-doping on the properties of (Lu,Gd)_2_O_3_:Eu transparent ceramics: Insight from the photoluminescent spectra in as-sintered and annealed state. Ceram. Int..

